# Frequencies of single nucleotide polymorphisms in genes regulating inflammatory responses in a community-based population

**DOI:** 10.1186/1471-2156-8-7

**Published:** 2007-03-14

**Authors:** Han-Yao Huang, Lucy Thuita, Paul Strickland, Sandra C Hoffman, George W Comstock, Kathy J Helzlsouer

**Affiliations:** 1 Department of Epidemiology, Johns Hopkins Bloomberg School of Public Health, Baltimore, Maryland, USA; 2 Department of Biostatistics & Epidemiology/Wb4, Cleveland Clinic Foundation, Cleveland, Ohio, USA; 3 Department of Environmental Health Sciences, Johns Hopkins School of Public Health, Baltimore, Maryland, USA; 4 Prevention and Research Center, Women's Center for Health & Medicine, Mercy Medical Center, Baltimore, Maryland, USA

## Abstract

**Background:**

Allele frequencies reported from public databases or articles are mostly based on small sample sizes. Differences in genotype frequencies by age, race and sex have implications for studies designed to examine genetic susceptibility to disease.

In a community-based cohort of 9,960 individuals, we compared the allele frequencies of 49 single nucleotide polymorphisms (SNPs) of genes involved in inflammatory pathways to the frequencies reported on public databases, and examined the genotypes frequencies by age and sex. The genes in which SNPs were analyzed include CCR2, CCR5, COX1, COX2, CRP, CSF1, CSF2, IFNG, IL1A, IL1B, IL2, IL4, IL6, IL8, IL10, IL13, IL18, LTA, MPO, NOS2A, NOS3, PPARD, PPARG, PPARGC1 and TNF.

**Results:**

Mean(SD) age was 53.2(15.5); 98% were Caucasians and 62% were women. Only 1 out of 33 SNPs differed from the SNP500Cancer database in allele frequency by >10% in Caucasians (n = 9,831), whereas 12 SNPs differed by >10% (up to 50%) in African Americans (n = 105). Two out of 15 SNPs differed from the dbSNP database in allele frequencies by >10% in Caucasians, and 5 out of 15 SNPs differed by >10% in African Americans. Age was similar across most genotype groups. Genotype frequencies did not differ by sex except for TNF(rs1799724), IL2(rs2069762), IL10(rs1800890), PPARG(rs1801282), and CRP(rs1800947) with differences of less than 4%.

**Conclusion:**

When estimating the size of samples needed for a study, particularly if a reference sample is used, one should take into consideration the size and ethnicity of the reference sample. Larger sample size is needed for public databases that report allele frequencies in non-Caucasian populations.

## Background

Chronic inflammation has been implicated in a wide variety of diseases, including cardiovascular disease, cancer, diabetes, neurological disorders, arthritis, among others [1-8]. Knowledge of genetic variations that influence inflammatory responses is important for assessing disease risk and identifying preventive strategies.

Several genes have been found to be involved in inflammatory responses. Examples are genes coding for interleukins (ILs) [9], tumor necrosis factors (TNFs) [9], cyclooxygenases (COX1 and COX2) [9], nitric oxide synthases (iNOS and eNOS) [10,11], peroxisome proliferator-activated receptors (PPARs) [12], CC chemokine receptors (CCR2, CCR5) [13,14], myeloperoxidase (MPO) [15], interferon-gamma (IFNG) [16], colony-stimulating factors (CSF1, CSF2) [17], lymphotoxin α (LTA) [18], and C-reactive protein (CRP) [18].

When designing a study to investigate genetic susceptibility to diseases, information on the allele frequencies of single nucleotide polymorphisms (SNPs) in the source population is crucial for ensuring sufficient statistical power. To date, the estimation for allele or genotype frequencies of candidate genes has been primarily based on limited numbers of individuals. For example, the SNP500Cancer database, a useful resource often used for referencing sequences and allele frequencies of validated SNPs, were based on 102 anonymous individuals with self-described heritage (24 African Americans, 31 Caucasians, 23 Hispanics, and 24 Pacific Rim heritages) [19]. On the dbSNP database, summary allele frequencies were calculated based on data from various ethnic groups, and often, several hundreds of samples were included [20]. The International HapMap Project analyzed 270 individuals, including 30 sets of samples from two parents and an adult child in Yoruba people of Ibadan, Nigeria, 45 unrelated individuals from Tokyo, 45 unrelated individuals from Beijing, and 30 U.S. trios with northern and western European ancestry [21]. Published studies of genetic polymorphisms have included no more than a few hundreds of individuals. The limited sample sizes make for uncertainties in estimating the allele frequencies in the general population.

Age and sex are often used as matching factors in epidemiological association studies because in most cases, they are associated with disease risk or survivorship. When genotype frequency is associated with age or sex, by matching on these factors in case-control studies, one would make the genotype frequency artificially similar between cases and controls. Under this circumstance, over-matching may occur.

To assess how allele frequencies reported on public databases are commensurate with the allele frequencies in the general population, we compared the allele frequencies of selected SNPs in candidate genes involved in inflammatory pathways in a large, community-based population in Washington County, Maryland to the allele frequencies reported from SNP500Cancer database, and if unavailable from SNP500Cancer, the dbSNP database. In addition, we compared the genotype frequencies among age and sex groups to explore whether overmatching by age or sex in an association study of genetic polymorphisms and disease risk may be of concern. The candidate genes included were CCR2, CCR5, COX1, COX2, CRP, CSF1, CSF2, IFNG, IL1A, IL1B, IL2, IL4, IL6, IL8, IL10, IL13, IL18, LTA, MPO, NOS2A, NOS3, PPARD, PPARG, PPARGC1 and TNF.

## Results

Characteristics of the study population, the Odyssey and CLUE II subcohort, were presented in Table 1. The Odyssey participants were older than the CLUE II subcohort, reflecting the fact that individuals in the Odyssey had participated in the CLUE I 15 years prior to CLUE II. The Odyssey cohort also had more women, higher body mass index (BMI), and more years of school education.

**Table 1:** Characteristics of the Odyssey and CLUE II subcohort study participants, Washington County, Maryland, 1989

**Characteristic**	**Total** **N = 9,960**	**Odyssey** **N = 8,307**	**Subcohort** **N = 2,460**	Age-adjusted OR (95%CI)*
**Age (yrs)**				
Mean (SD)	53.2 (15.5)	55.6 (13.5)	45.7 (18.5)	1.04(1.04,1.05)^†^
Min, Max	5, 95	28, 95	5, 94	
Median (IQR)	54 (42, 65)	56 (45, 66)	46 (32, 61)	
**Gender**				
Male	3829 (38)	3111 (37)	1035 (42)	1.0
Female	6131 (62)	5196 (62)	1425 (58)	1.22(1.11,1.35)
**Race**				
White	9831 (99)	8213 (99)	2415 (98)	1.0
Black	105 (1)	81 (1)	34 (1)	0.95(0.63,1.44)
Others	24	13	11	0.48(0.21,1.14)
**BMI (kg/m**^2^**)**				
< 25	4269 (43)	3392 (41)	1219 (50)	1.0
25 – 29.9	3758 (38)	3259 (39)	794 (32)	1.26(1.13,1.40)
=> 30	1921 (19)	1647 (20)	444 (18)	1.26(1.11,1.43)
Missing	12	9	3	
**Cigarette smoking**				
Never	5363 (54)	4474 (54)	1318 (53)	1.0
Former	2952 (30)	2545 (31)	669 (27)	0.90(0.81,1.01)
Current	1645 (17)	1288 (15)	473 (19)	0.91(0.80,1.04)
**Education (years)**				
<12	2476 (25)	1994 (24)	680 (28)	1.0
=12	4555 (46)	3869 (47)	1038 (42)	1.65(1.47,1.86)
>12	2923 (29)	2439 (29)	741 (30)	1.50(1.32,1.71)
Missing	6	5	1	
**Blood pressure**^‡^				
Normal	2277 (23)	1719 (21)	705 (29)	1.0
Prehypertensive	4471(45)	3684 (44)	1159 (47)	0.95(0.84,1.07)
Hypertensive	3204 (32)	2896 (35)	595 (24)	0.95(0.83,1.09)
Missing	8	8	1	
**Cholesterol(mg/dl) with no treatment**				
< 200	4092 (44)	3223 (42)	1180 (50)	1.0
200 – 239	3472 (37)	2979 (38)	791 (33)	1.00(0.89,1.11)
>= 240	1773 (19)	1536 (20)	386 (16)	0.93(0.81,1.07)
**Cholesterol(mg/dl) with treatment**				
< 200	119 (23)	108 (22)	21 (25)	1.0
200 – 239	223 (42)	205 (43)	33 (39)	1.19(0.65,2.15)
>= 240	184 (35)	168 (35)	30 (36)	1.05(0.57,1.93)

* age adjusted odds ratio of being an Odyssey cohort participant.

^† ^per year increment.

^‡ ^Normal blood pressure was defined as individuals with systolic pressure <120 mmHg and diastolic blood pressure of 80 mmHg and not on anti-hypertensive medication; Hypertension was defined as under anti-hypertensive medication or systolic pressure >140 mmHg or diastolic pressure of 90 mmHg. Individuals with systolic blood pressure between 120 and 140 mmHg and/or diastolic blood pressure between 80 mmHg and 90 mmHg were considered pre-hypertensive.

Among the 33 SNPs that were both reported by the present study and the SNP500 Cancer Database, the allele frequency of only 1 SNP differed by more than 10% in Caucasians, whereas the allele frequencies of 7 SNPs differed by 11–20%, 4 SNPs differed by 21–30%, and 1 SNP differed by 50% in African Americans (Table 2, see Additional file 1). Among the 15 SNPs that were also reported on the dbSNP database, 2 SNPs differed by more than 10–20% in Caucasians, whereas in African Americans, the allele frequencies of 5 SNPs differed by 10–20%. Most SNPs followed the Hardy-Weinberg equilibrium with the exceptions of CCR5 (rs333), COX1 (rs3842787), CRP (rs1800947), CSF2 (rs1469149), IL1B (rs16944), IL4 (rs2243250), IL18 (rs187238), LTA (rs2857713), LTA (rs3093543), and NOS2A (rs2297518) (Table 2; see Additional file 1).

Age was similar among the genotype groups with a few exceptions (Table 3; see Additional file 2). Specifically, statistically significant differences of one to three years of age were observed for the genotypes of TT vs. CC of IL4 (rs2243250), AT vs. TT of IL10 (rs1800890), AG vs. GG of IL10 (rs1800896), AG vs. AA of NOS2A (rs2297518), and GG vs. AA of PPARG (rs709158). Genotype frequencies did not differ by sex except for TNF (rs1799724), IL2 (rs2069762), IL10 (rs1800890), PPARG (rs1801282), and CRP (rs1800947) with differences of no more than 4% between two groups (Table 4; see Additional file 3).

## Discussion

We report allele frequencies and genotype frequencies in a large community-based population, predominantly of Caucasians. Although the CLUE cohorts were not enrolled through a random sampling process, we find no particular reason to suggest that the genetic composition of CLUE participants would have affected research participation. This notion is supported by the findings that the frequency distributions of genetic polymorphisms did not differ between the Odyssey and the CLUE II subcohort.

The similar allele frequencies in CLUE's Caucasians to the frequencies reported from the SNP500Cancer database have implications for the design of studies on genetic polymorphisms. When the SNP500Cancer database is used as a source of reference for SNP selection with allele frequencies as one of the selection criteria, and if both wild-type and variant alleles have fairly high frequencies (30%–70%), even a discrepancy in allele frequency between study samples and the samples used in the SNP500Cancer project is up to 20% may not influence investigators' decision on including into a particular SNP into a study. However, for rarer alleles, sampling errors resulting in variations in allele frequencies estimates can have an impact on SNP selection. For example, we chose to study a SNP (rs1726803) of POLD1 gene and a SNP (rs6413413) of ADH2 gene that were reported on the SNP500Cancer database to have a minor allele frequency of 5%. After analyzing approximately 3,000 samples, we found no variation in the SNP allele frequency and stopped this genotyping analysis.

Among the 49 SNPs examined, 10 SNPs did not follow the Hardy-Weinburg equilibrium. Genotyping is not 100% accurate and failures to call out genotypes might have been a reason for the H-W disequilibrium. On the other hand, the sample size is fairly large in this study, and the larger the sample size is, the easier for any discrepancy in the observed allele frequency from the expected frequency (according to an H-W equilibrium) to reach statistical significance.

Although in the present study, the number of African American participants was limited (n = 105), it exceeded the size reported on the SNP500Cancer databases (n = 24). As expected, there were greater differences in the allele frequencies between the present study and the SNP500Cancer database for African Americans; allele frequencies significantly differed for 35% of the SNPs for which comparisons could be made. This finding raises concerns about the usefulness of the SNP500Cancer database as a reference for candidate gene selection for African Americans.

We observed sex- or age-differences in genotype frequencies for some of the SNPs. Chance alone cannot be excluded from being a possible explanation for the statistically significant differences in genotype frequencies between age groups or sex groups, particularly because the differences in genotypes between age and sex groups were small in the present study. Replication is needed for testing the robustness of these findings.

## Conclusion

In conclusion, when estimating the size of samples needed for a study, particularly if a reference sample is used, one should take into consideration the size and the ethnicity of the reference sample. The greater differences in allele frequencies among African Americans between the present study and public databases indicate a need for basing public databases on a larger sample size for this ethnic group. The small differences in genotype frequencies by age or sex for some candidate genes may be explained by chance alone, and more published data are needed for replication.

## Methods

### Study population

The study population consists of participants in two community-based cohorts, CLUE I and CLUE II, in Washington County, Maryland. Washington County has a slowly growing population. The majority (98%) of the Washington County residents is Caucasian; 1.5% are African Americans. CLUE I (from a slogan, give us a CLUE to cancer and heart disease) was established in 1974 for the purposes of collecting brief questionnaire data and setting up a serum bank for research purposes. Participants were recruited at mobile trailers that were stationed in various locations in Washington County to offer opportunities for research participation. A total of 26,147 individuals participated in CLUE I. Of these, 23,951 were Washington County residents. Participation was better in women, in those who had more years of education and in the age group of 45–64 years.

The CLUE II was a similar campaign conducted in 1989 in which plasma samples, buffy coat samples and information on participants' selected characteristics and diet were collected. A total of 32,896 individuals participated in CLUE II, and of these, 25,079 were Washington County residents. Both CLUE I and CLUE II cohorts included approximately 30% of the County residents. The racial distributions of the CLUE I and CLUE II cohorts were similar to that in the Washington County. CLUE II participation was slightly better among women, among those who had more years of school education, and among those who were at age of 45–70 years.

The present study includes the Odyssey cohort and a CLUE II subcohort. The Odyssey cohort is composed of the 8,394 individuals who took part in both CLUE I and CLUE II. Within Odyssey, DNA was successfully extracted from the buffy coat samples of 8,307 individuals. The CLUE II subcohort (n = 2,460) is an age- and sex-stratified random sample (10%) of the CLUE II participants who were residents of Washington County and its neighboring areas. DNA samples from this subcohort were successfully extracted from all buffy coat samples. Among these, 807 samples overlapped with the 8,307 Odyssey samples. Hence, 9960 samples are included in the present study.

SNP data were rendered anonymous by in-house Anonymous Data Management software. The study identifiers were encrypted by modern cryptographic technologies that destroyed the link from genetic information back to identities, while anonymously allowing one-way linkage from identities to genetic information. Informed content to take part in the CLUE I and CLUE II research campaign was obtained from each participant. The protocol was approved by the Institutional Review Board of the Johns Hopkins Bloomberg School of Public Health.

### Selection of candidate genes

Candidate genes were selected based on the following criteria: (a) estimated allele frequencies of ≥ 5% in Caucasians in published literature or databases, (b) known or promising importance in the development of cancer, cardiovascular diseases, and/or longevity, (c) validated allele substitutions, and/or (d) functional changes linked to allele substitutions that have been published in the literature.

### Laboratory analysis

At CLUE II enrollment, blood samples were collected into 20-ml heparinized Vacutainers (Fisher Scientific, Pittsburgh, PA). Samples were refrigerated at 4°C and most samples were centrifuged within 2 to 6 hours after blood collection and were never 24 hours later. Plasma aliquots from each participant were placed in two 5-ml Cryotubes (Sumitomo Bakelite, Neptune, NJ) and were stored at -70°C. Buffy coat samples were stored in separate vials at -70°C until extraction. Barcoding was performed as part of the blood collection process. Labels were printed with the study numbers barcoded so that they could be scanned for accuracy in data entry and inventory maintenance.

DNA was extracted from buffy coat by the alkaline lysis method [22]. Following isolation, DNA samples were resuspended in 10 mM Tris-HCl/1 mM EDTA (TE) and DNA concentration was adjusted to 100 μg/ml. Genotyping was performed by Celera Genomics Co. (Rockville, DC) for the 17 SNPs with rs numbers 1799864, 333, 1800629, 1800587, 1143634, 16944, 2243250, 1800795, 4073, 1800871, 1800872, 2143416, 2745557, 2206593, 4684847, 709158, and 1175543, and subsequently by Applied Biosystems Inc. (Foster City, CA) for the 32 SNPs with rs numbers 3842787, 5275, 1205, 1800947, 1130864, 2794521, 105885, 1469149, 25882, 2069705, 17561, 2069762, 1800797, 1800890, 1800896, 20541, 1800925, 187238, 1946518, 2857713, 3093543, 1041981, 909253, 2243828, 2333227, 2297518, 1799983, 2016520, 1801282, 8192678, 1799724, and 1799964. Both laboratories used the *Taq*Man assay.

Assay reliability was assessed for the 17 SNPs analyzed by Celera, using 86 duplicate samples randomly inserted into the plates; the percent concordance was 82–95%. Two SNPs (rs 1800587 and rs 17561) were both analyzed by Celera and ABI. The percent concordance was 0.89 and 0.90, respectively.

### Statistical analysis

Characteristics of the Odyssey and the CLUE II subcohort study participants were summarized by the frequency distributions of categorical variables and by the means (SDs) of continuous variables. The genotype frequencies of each candidate gene were compared between the Odyssey and the CLUE II subcohort. For most SNPs, no differences in genotype frequencies were observed between the Odyssey and the subcohort. The only exceptions were LTA (rs3093543) for which a higher proportion of AC as compared to AA genotype was observed [OR = 1.18 (1.05, 1.32)] and PPARGC1 (rs8192678) for which a higher proportion of AA as compared to GG genotype was observed [OR = 1.28 (1.08, 1.51)] in the Odyssey compared to the subcohort. Subsequent analyses were performed on the combined Odyssey and subcohort data.

Allele frequencies were examined separately in Caucasians and African Americans, and were compared to the frequencies reported from the SNP500Cancer database or if unavailable from SNP500Cancer, the dbSNP database. Statistically significant differences were reported if the allele frequencies from public databases fell beyond the 95% confidence intervals (95% CIs) of the allele frequencies among the CLUE population. The Hardy-Weinberg equilibrium was tested by a goodness-of-fit approach. Genotype frequencies by age groups of 10-year intervals were examined. Because age as a continuous variable provides more information, the One-Way Analysis Of Variance (ANOVA), rather than χ^2 ^tests for age categories by genotypes, was used to compare age as continuous variable across the genotype categories. Genotype frequencies were compared between men and women by χ^2 ^test.

## Abbreviations

IL: interleukins; TNFs: tumor necrosis factors; COX: cyclooxygenase; NOS: nitric oxide synthase; PPARs: peroxisome proliferator-activated receptors; CCR: chemokine receptors, MPO: myeloperoxidase; IFNG: interferon-gamma; CSF: colony-stimulating factors; LTA: lymphotoxin alpha; CRP: C-reactive protein.

## Authors' contributions

HYH conceived the study aims, participated in study design and statistical analysis, interpreted the data and drafted the manuscript. LT performed statistical analysis. PS participated in study design and interpreted the data. SCH participated in study coordination and data management. GWC helped to interpret the data. KJH participated in study design and interpreted the data. All authors read and approved the final manuscript.

## Supplementary Material

Additional File 1 Table 2. Allele frequencies of selected SNPs in the CLUE II study and the SNP500Cancer database; description: List of allele frequencies of selected SNPs involved in inflammatory pathways observed on CLUE II study samples and on the SNP500 Cancer database, and tests for H-W equilibrium. Click here for file

Additional File 2 Table 3. Genotype frequencies by age groups in Odyssey and CLUE II subcohort; description: Genotype distribution by age of 10 years intervals in the study samples. Click here for file

Additional File 3 Table 4. Genotype frequencies by sex in Odyssey and CLUE II subcohort; description: Genotype frequencies in men and women of the combined Odyssey and CLUE II subcohort. Click here for file
