# Supplementary material for: Frequencies of single nucleotide polymorphisms in genes regulating inflammatory responses in a community-based population
Source: BMC Genet. 2007 Mar 14;8:7. doi: 10.1186/1471-2156-8-7 (PMC1838428; doi:10.1186/1471-2156-8-7)
Supplement: Additional File 1 — Table 2. Allele frequencies of selected SNPs in the CLUE II study and the SNP500Cancer database; description: List of allele frequencies of selected SNPs involved in inflammatory pathways observed on CLUE II study samples and on the SNP500 Cancer database, and tests for H-W equilibrium. [file 1471-2156-8-7-S1.doc]

**Table 2. Allele frequencies of selected SNPs in the CLUE II study and the SNP500Cancer database**

| **Gene** | **Symbol** | **SNP** | **Major/minor**  **allele** | **Allele frequency in**  **Caucasians (n=9,831)**  **CLUE SNP500Cancer**  **or dbSNP*** | | **Difference**  **(95% CI)** | **Allele frequency in African American (n=105)**  **CLUE SNP500Cancer**  **or dbSNP*** | | **Difference**  **(95% CI)** | **H-W equili-brium**  **(p-value)^†^** |
| --- | --- | --- | --- | --- | --- | --- | --- | --- | --- | --- |
| chemokine (C-C motif) receptor 2 | CCR2 | rs1799864 | G/A | 0.92(0.92,0.93) | 0.87 ^‡^ | 0.05(0.04,0.05) | 0.85(0.78,0.92) | 0.83 | 0.02(–0.007,0.05) | 0.34 |
| chemokine (C-C motif) receptor 5 | CCR5 | rs333 | G/– | 0.89(0.88,0.90) | 0.95 ^‡^ | –0.06(–0.06,–0.05) | 0.93(0.88,0.98) | 1.0 ^‡^ | –0.07(–0.12,–0.02) | 0.001 |
| prostaglandin-endoperoxide synthase 1 | COX1 | rs3842787 | C/T | 0.93(0.92,0.94) | 0.91^*‡^ | 0.02(0.02,0.02) | 0.86(0.79,0.93) | 0.91^*^ | –0.05(–0.09,–0.01) | 0.03 |
| prostaglandin-endoperoxide synthase 2 | COX2 | rs5275 | T/C | 0.66(0.65,0.67) | 0.65 | 0.01(0.008,0.01) | 0.57(0.48,0.66) | 0.33 ^‡^ | 0.24(0.16,0.32) | 0.29 |
| prostaglandin-endoperoxide synthase 2 | COX2 | rs2143416 | A/C | 0.84(0.83,0.85) | 0.77 ^*‡^ | 0.07(0.06,0.07) | 0.57(0.48,0.66) | 0.77 ^*‡^ | –0.20(–0.28,–0.12) | 0.34 |
| prostaglandin-endoperoxide synthase 2 | COX2 | rs2206593 | C/T | 0.94(0.94,0.94) | 0.95 ^*‡^ | –0.01(–0.01,–0.008) | 0.99(0.97,1.00) | 0.95 ^*‡^ | 0.04(0.002,0.08) | 0.75 |
| prostaglandin-endoperoxide synthase 2 | COX2 | rs2745557 | C/T | 0.82(0.81,0.83) | 0.82 ^*^ | 0 | 0.84(0.77,0.91) | 0.82 ^*^ | 0.02(–0.007,0.05) | 0.84 |
| C-reactive protein | CRP | rs1205 | C/T | 0.67(0.66,0.68) | 0.69 ^‡^ | –0.02(–0.02,–0.017) | 0.77(0.69,0.85) | 0.90 ^‡^ | –0.13(–0.19,–0.06) | 0.51 |
| C-reactive protein | CRP | rs1800947 | C/G | 0.94(0.94,0.94) | 0.94 | 0 | 0.99(0.97,1.00) | 1.0 | –0.01(–0.03,0.01) | <0.0001 |
| C-reactive protein | CRP | rs1130864 | C/T | 0.69(0.68,0.70) | 0.87 ^*‡^ | –0.18(–0.19,–0.17) | 0.83(0.76,0.90) | 0.87 ^*^ | –0.04(–0.08,0.0) | 0.23 |
| C-reactive protein | CRP | rs2794521 | T/C | 0.72(0.71,0.73) | 0.79 ^*‡^ | –0.07(–0.06,–0.075) | 0.80(0.72,0.88) | 0.79 ^*^ | 0.01(–0.01,0.03) | 0.17 |
| colony stimulating factor 1 (macrophage) | CSF1 | rs1058885 | T/C | 0.67(0.66,0.68) | 0.83 ^*‡^ | –0.17(–0.18,–0.16) | 0.73(0.65,0.81) | 0.83 ^*‡^ | –0.10(–0.16,–0.04) | 0.64 |
| colony stimulating factor 2 (granulocyte-macrophage) | CSF2 | rs1469149 | A/C | 0.66(0.65,0.67) | 0.65 | 0.01(0.008,0.012) | 0.73(0.65,0.81) | 0.57 ^‡^ | 0.16(0.09,0.23) | 0.04 |
| colony stimulating factor 2 (granulocyte-macrophage) | CSF2 | rs25882 | T/C | 0.79(0.78,0.80) | 0.76 ^‡^ | 0.03(0.027,0.03) | 0.75(0.67,0.83) | 0.75 | 0 | 0.67 |
| interferon, gamma | IFNG | rs2069705 | A/G | 0.68(0.67,0.69) | 0.58 ^‡^ | 0.10(0.09,0.11) | 0.56(0.47,0.65) | 0.50 | 0.06(0.02,0.11) | 0.93 |
| interleukin 1, alpha | IL1A | rs17561 | C/A | 0.71(0.70,0.72) | 0.65 ^‡^ | 0.06(0.055,0.06) | 0.81(0.73,0.89) | 0.77 | 0.04(0.002,0.08) | 0.31 |
| interleukin 1, alpha | IL1A | rs1800587 | A/G | 0.72(0.71,0.73) | 0.64 ^‡^ | 0.08(0.07,0.08) | 0.61(0.52,0.70) | 0.61 | 0 | 0.12 |
| interleukin 1, beta | IL1B | rs16944 | C/T | 0.67(0.66,0.68) | 0.74 ^‡^ | –0.08(–0.08,0.07) | 0.52(0.42,0.62) | 0.42 | 0.10(0.04,0.16) | <0.0001 |
| interleukin 1, beta | IL1B | rs1143634 | C/T | 0.70(0.69,0.71) | 0.68 ^‡^ | 0.02(0.02,0.02) | 0.84(0.77,0.91) | 0.85 | –0.01(–0.03,0.01) | 0.06 |
| interleukin 2 | IL2 | rs2069762 | A/C | 0.70(0.69,0.71) | 0.69 | 0.01(0.008,0.01) | 0.87(0.81,0.93) | 0.92 | –0.05(–0.09,–0.01) | 0.99 |
| interleukin 4 | IL4 | rs2243250 | C/T | 0.86(0.85,0.87) | 0.90 ^‡^ | –0.04(–0.044,–0.036) | 0.47(0.37,0.57) | 0.21 ^‡^ | 0.26(0.18,0.34) | <0.0001 |
| interleukin 6 | IL6 | rs1800797 | G/A | 0.66(0.65,0.67) | 0.52 ^‡^ | 0.14(0.13,0.15) | 0.54(0.44,0.64) | 0.04 ^‡^ | 0.50(0.40,0.60) | 0.84 |
| interleukin 6 | IL6 | rs1800795 | G/C | 0.57(0.56,0.58) | 0.50 ^‡^ | 0.07(0.06,0.07) | 0.93(0.88,0.98) | 0.96 | –0.03(–0.06,0.0) | 0.95 |
| interleukin 8 | IL8 | rs4073 | T/A | 0.55(0.54,0.56) | 0.57 ^‡^ | –0.02(–0.023,–0.02) | 0.26(0.18,0.34) | 0.21 | 0.05(0.01,0.09) | 0.73 |
| interleukin 10 | IL10 | rs1800871 | C/T | 0.76(0.75,0.77) | 0.71 ^‡^ | 0.05(0.046,0.054) | 0.63(0.54,0.72) | 0.50 ^‡^ | 0.13(0.06,0.19) | 0.13 |
| interleukin 10 | IL10 | rs1800872 | C/A | 0.76(0.75,0.77) | 0.71 ^‡^ | 0.05(0.046,0.054) | 0.63(0.54,0.72) | 0.50 ^‡^ | 0.13(0.06,0.19) | 0.41 |
| interleukin 10 | IL10 | rs1800890 | A/T | 0.66(0.65,0.67) | 0.70 ^‡^ | –0.04(–0.044,–0.036) | 0.67(0.58,0.76) | 0.88 | –0.21(–0.29,–0.13) | 0.57 |
| interleukin 10 | IL10 | rs1800896 | A/G | 0.63(0.62,0.64) | 0.60 ^‡^ | 0.03(0.027,0.033) | 0.73(0.65,0.81) | 0.67 | 0.06(0.01,0.11) | 0.33 |
| interleukin 13 | IL13 | rs20541 | G/A | 0.81(0.80,0.82) | 0.86 ^‡^ | –0.05(–0.054,–0.046) | 0.85(0.78,0.92) | 0.88 | –0.03(–0.06,0.0) | 0.13 |
| interleukin 13 | IL13 | rs1800925 | C/T | 0.80(0.79,0.81) | 0.86 ^‡^ | –0.06(–0.064,–0.055) | 0.73(0.65,0.81) | 0.46 ^‡^ | 0.27(0.19,0.35) | 0.49 |
| interleukin 18 | IL18 | rs187238 | C/G | 0.73(0.72,0.74) | 0.80 ^*‡^ | –0.07(–0.075,–0.065) | 0.77(0.69,0.85) | 0.80 ^*^ | –0.03(–0.06,0.0) | 0.03 |
| interleukin 18 | IL18 | rs1946518 | G/T | 0.66(0.65,0.67) | 0.60 ^*‡^ | 0.06(0.05,0.06) | 0.72(0.63,0.81) | 0.60 ^*‡^ | 0.12(0.06,0.18) | 0.26 |
| lymphotoxin alpha | LTA | rs2857713 | T/C | 0.73(0.72,0.74) | 0.75 ^*‡^ | –0.02(–0.02,–0.017) | 0.74(0.66,0.82) | 0.75 ^*^ | –0.01(–0.03,0.01) | 0.001 |
| lymphotoxin alpha | LTA | rs3093543 | A/C | 0.93(0.92,0.94) | 0.95 ^*‡^ | –0.02(–0.02,–0.017) | 0.99(0.97,1.00) | 0.95 ^*‡^ | 0.04(0.0,0.08) | <0.0001 |
| lymphotoxin alpha | LTA | rs1041981 | C/A | 0.66(0.65,0.67) | 0.59 ^*‡^ | 0.07(0.06,0.07) | 0.50(0.40,0.60) | 0.59 ^*^ | –0.09(–0.14,–0.04) | 0.09 |
| lymphotoxin alpha | LTA | rs909253 | T/C | 0.66(0.65,0.67) | 0.76 ^‡^ | –0.10(–0.11,–0.09) | 0.65(0.59,0.74) | 0.54 ^‡^ | 0.11(0.05,0.17) | 0.08 |
| Myeloperoxidase | MPO | rs2243828 | A/G | 0.78(0.77,0.79) | 0.83 ^‡^ | –0.05(–0.054,–0.046) | 0.69(0.60,0.78) | 0.50 ^‡^ | 0.19(0.11,0.26) | 0.19 |
| Myeloperoxidase | MPO | rs2333227 | C/T | 0.79(0.78,0.80) | 0.83 ^‡^ | –0.04(–0.044,–0.036) | 0.68(0.59,0.77) | 0.50 ^‡^ | 0.18(0.11,0.25) | 0.14 |
| nitric oxide synthase 2A | NOS2A | rs2297518 | G/A | 0.81(0.80,0.82) | 0.87 ^‡^ | –0.06(–0.065,–0.055) | 0.87(0.81,0.93) | 0.85 | 0.02(–0.01,0.05) | 0.03 |
| nitric oxide synthase 3 | NOS3 | rs1799983 | G/T | 0.67(0.66,0.68) | 0.67 | 0 | 0.86(0.79,0.93) | 0.92 | –0.06(–0.11,–0.01) | 0.42 |
| peroxisome proliferator-activated receptor delta | PPARD | rs2016520 | T/C | 0.81(0.80,0.82) | 0.85 ^‡^ | –0.04(–0.044,–0.036) | 0.75(0.67,0.83) | 0.77 ^‡^ | –0.02(–0.05,0.01) | 0.61 |
| peroxisome proliferator-activated receptor gamma | PPARG | rs709158 | A/G | 0.64(0.63,0.65) | 0.74 ^*‡^ | –0.10(–0.106,–0.09) | 0.88(0.82,0.94) | 0.74 ^*^ | 0.14(0.07,0.21) | 0.17 |
| peroxisome proliferator-activated receptor gamma | PPARG | rs1175543 | A/G | 0.64(0.63,0.65) | 0.75 ^*‡^ | –0.09(–0.096,–0.08) | 0.88(0.82,0.94) | 0.75 ^*‡^ | 0.13(0.06,0.19) | 0.1 |
| peroxisome proliferator-activated receptor gamma | PPARG | rs1801282 | C/G | 0.89(0.88,0.90) | 0.89 | 0 | 0.97(0.94,1.00) | 0.96 | 0.01(–0.01,0.03) | 0.08 |
| peroxisome proliferator-activated receptor gamma | PPARG | rs4684847 | C/T | 0.89(0.88,0.90) | N/A | N/A | 0.96(0.92,1.00) | N/A | N/A | 0.16 |
| peroxisome proliferator-activated receptor gamma, coactivator 1 alpha | PPARGC1A | rs8192678 | G/A | 0.66(0.65,0.67) | 0.71 ^*‡^ | –0.05(–0.054,–0.046) | 0.88(0.82,0.94) | 0.71 ^*‡^ | 0.17(0.10,0.24) | 0.19 |
| tumor necrosis factor | TNF | rs1799724 | C/T | 0.91(0.90,0.92) | 0.90 | 0.01(0.008,0.012) | 0.99(0.97,1.00) | 0.96 ^‡^ | 0.03(0.0,0.06) | 0.29 |
| tumor necrosis factor | TNF | rs1799964 | T/C | 0.78(0.77,0.79) | 0.75 ^‡^ | 0.03(0.027,0.033) | 0.87(0.81,0.93) | 0.80 | 0.07(0.02,0.12) | 0.06 |
| tumor necrosis factor | TNF | rs1800629 | G/A | 0.84(0.83,0.85) | 0.83 | 0.01(0.008,0.012) | 0.86(0.79,0.93) | 0.92 | 0.06(0.01,0.11) | 0.72 |

^*^  allele frequencies from the dbSNP database

^†^ test for the Hardy-Weinberg equilibrium for CLUE samples.

^‡^ P<0.05 for the comparison on the allele frequencies between CLUE and public databases.

N/A: data unavailable from SNP500Cancer or dbSNP databases
