# Supplementary material for: Frequencies of single nucleotide polymorphisms in genes regulating inflammatory responses in a community-based population
Source: BMC Genet. 2007 Mar 14;8:7. doi: 10.1186/1471-2156-8-7 (PMC1838428; doi:10.1186/1471-2156-8-7)
Supplement: Additional File 2 — Table 3. Genotype frequencies by age groups in Odyssey and CLUE II subcohort; description: Genotype distribution by age of 10 years intervals in the study samples. [file 1471-2156-8-7-S2.doc]

**Table 3. Genotype frequencies by age groups in Odyssey and CLUE II subcohort**

| **Gene** | **SNP** | **Genotype (%)** | **Mean (SD)** | **Age<30**  **n=639** | **30-39**  **n=1,352** | | **40-49**  **n=1,999** | | **50-59**  **n=2,112** | | **60-69**  **n=2,362** | | **70-79**  **n=1,268** | | **80+**  **n=228** | |
| --- | --- | --- | --- | --- | --- | --- | --- | --- | --- | --- | --- | --- | --- | --- | --- | --- |
| CCR2 | rs1799864 | GG  GA  AA  Missing | 52.9 (15.5)  53.4 (15.5)  54.0 (16.3)  56.0 (14.5) | 82  14  1  3 | 80  14  0.5  5 | | 80  14  0.6  5 | | 79  14  6  0.7 | | 79  14  6  0.8 | | 77  15  7  0.8 | | 74  18  8  0 | |
| CCR5 | rs333 | GG  G-  --  Missing | 53.2 (15.4)  52.9 (15.6)  52.5 (16.3)  53.5 (15.8) | 74  17  2  7 | 74  18  1  7 | | 76  17  1  6 | | 75  17  1  6 | | 74  18  2  6 | | 75  16  1  8 | | 75  17  1  7 | |
| COX1 | rs3842787 | CC  CT  TT  Missing | 52.8 (15.5)  52.8 (15.0)  55.1 (12.1)  57.8 (15.7) | 85  10  0  4 | 85  11  1  3 | | 84  13  0  3 | | 82  13  1  4 | | 82  11  1  6 | | 81  10  0  8 | | 79  10  0  10 | |
| COX2 | rs5275 | TT  CT  CC  Missing | 53.1 (15.2)  52.6 (15.7)  52.2 (15.3)  57.4 (15.0) | 38  45  13  4 | 41  44  11  4 | | 42  42  11  5 | | 42  40  12  6 | | 39  41  12  8 | | 41  41  9  9 | | 39  42  7  12 | |
| COX2 | rs2143416 | AA  AC  CC  Missing | 53.2 (15.5)  52.6 (15.4)  51.6 (15.2)  55.6 (15.4) | 67  26  4  3 | 65  27  3  4 | | 65  28  3  4 | | 68  24  2  5 | | 66  26  3  5 | | 67  25  2  6 | | 68  25  1  7 | |
| COX2 | rs2206593 | CC  CT  TT  Missing | 53.0 (15.4)  53.4 (15.6)  56.5 (12.7)  54.5 (16.3) | 85  10  0  4 | | 86  11  0.2  3 | | 84  11  0.6  3 | | 85  10  0.4  4 | | 85  10  0.3  4 | | 83  11  0.5  5 | | 81  13  0.4  6 |
| COX2 | rs2745557 | CC  CT  TT  Missing | 52.9 (15.5)  53.3 (15.5)  54.9 (14.0)  54.7 (15.8) | 67  28  2  4 | | 66  27  2  4 | | 64  29  3  4 | | 64  27  3  5 | | 66  27  3  4 | | 62  29  3  6 | | 60  30  2  8 |
| CRP | rs1205 | CC  CT  TT  Missing | 52.6 (15.5)  52.9 (15.3)  53.4 (15.4)  58.1 (15.6) | 43  42  10  4 | | 47  41  10  3 | | 44  42  10  3 | | 43  42  10  4 | | 42  42  11  5 | | 42  40  10  8 | | 43  37  10  10 |
| CRP | rs1800947 | CC  CG  GG  Missing | 52.7 (15.3)  52.6 (15.5)  56.7 (14.5)  55.2 (15.9) | 77  9  0  13 | | 82  8  0  10 | | 79  10  1  11 | | 78  9  1  12 | | 76  9  1  15 | | 73  9  1  17 | | 76  6  1  16 |
| CRP | rs1130864 | CC  CT  TT  Missing | 52.8 (15.5)  52.7 (15.3)  53.0 (15.4)  57.7 (15.5) | 47  39  9  5 | | 46  41  10  3 | | 46  41  9  4 | | 44  41  10  5 | | 45  39  8  8 | | 44  37  10  8 | | 44  34  10  12 |
| CRP | rs2794521 | TT  CT  CC  Missing | 53.0 (15.3)  52.5 (15.5)  53.0 (15.3)  57.6 (15.6) | 49  39  8  4 | | 50  40  7  3 | | 50  39  8  3 | | 51  37  7  5 | | 49  37  8  6 | | 50  34  8  8 | | 45  36  8  11 |
| CSF1 | rs1058885 | TT  CT  CC  Missing | 52.7 (15.4)  52.6 (15.6)  53.0 (15.2)  57.8 (14.9) | 34  49  13  5 | | 39  45  12  4 | | 38  43  14  5 | | 36  44  13  6 | | 35  45  12  8 | | 37  40  14  10 | | 35  43  11  11 |
| CSF2 | rs1469149 | AA  AC  CC  Missing | 53.1 (15.5)  53.1 (15.4)  52.4 (15.6)  55.9 (15.6) | 33  48  16  3 | | 34  48  17  2 | | 33  48  17  2 | | 33  48  16  3 | | 32  49  15  3 | | 34  47  16  3 | | 33  48  12  6 |
| CSF2 | rs25882 | TT  CT  CC  Missing | 52.7 (15.6)  52.8 (15.1)  53.6 (15.5)  57.7 (15.2) | 62  29  3  5 | | 60  32  5  3 | | 60  32  3  5 | | 58  32  4  6 | | 58  31  4  8 | | 57  29  4  10 | | 56  28  4  11 |
| IFGN | rs2069705 | AA  AG  GG  Missing | 53.0 (15.3)  52.6 (15.5)  52.5 (15.4)  57.4 (15.4) | 40  45  10  5 | | 45  41  10  4 | | 43  41  10  5 | | 42  41  11  6 | | 43  41  8  8 | | 41  39  10  10 | | 44  34  8  15 |
| IL1A | rs1800587 | GG  AG  AA  Missing | 53.1 (15.5)  52.8 (15.3)  51.8 (15.6)  57.5 (15.0) | 50  38  8  4 | | 50  38  9  3 | | 49  40  8  3 | | 48  39  8  5 | | 49  38  8  6 | | 50  36  7  7 | | 52  33  8  8 |
| IL1A | rs17561 | CC  AC  AA  Missing | 53.0 (15.5)  52.9 (15.3)  51.7 (15.6)  57.3 (14.9) | 50  38  8  3 | | 51  37  9  3 | | 49  40  8  4 | | 48  39  8  4 | | 49  38  8  5 | | 49  37  6  7 | | 50  34  7  8 |
| IL1B | rs16944 | CC  CT  TT  Missing | 53.0 (15.5)  52.5 (15.7)  53.2 (15.2)  55.8 (14.8) | 41  42  10  7 | | 41  38  11  10 | | 41  38  11  10 | | 39  36  11  13 | | 41  36  11  12 | | 40  35  11  14 | | 35  35  10  20 |
| IL1B | rs1143634 | CC  CT  TT  Missing | 52.8 (15.3)  52.6 (16.0)  52.6 (15.3)  56.0 (14.7) | 53  35  4  7 | | 54  31  5  11 | | 54  29  5  12 | | 52  30  5  13 | | 52  28  5  15 | | 50  29  4  17 | | 46  32  3  19 |
| IL2 | rs2069762 | AA  CA  CC  Missing | 53.1 (15.6)  52.3 (15.3)  53.3 (15.4)  57.6 (15.3) | 46  41  9  4 | | 49  40  8  3 | | 47  41  8  4 | | 46  41  8  5 | | 46  39  9  7 | | 48  34  9  9 | | 51  32  7  10 |
| IL4^*^ | rs2243250 | CC  CT  TT  Missing | 53.1 (15.5)  53.2 (15.5)  50.1 (14.7)  54.6 (15.3) | 70  23  2  5 | | 70  21  3  6 | | 69  22  3  6 | | 69  21  3  7 | | 69  23  2  6 | | 70  21  2  7 | | 69  21  1  9 |
| IL6 | rs1800797 | GG  GA  AA  Missing | 53.0 (15.4)  52.9 (15.6)  53.0 (15.0)  57.1 (15.2) | 32  49  16  2 | | 33  50  16  2 | | 37  44  17  2 | | 32  48  17  3 | | 34  46  17  3 | | 34  47  15  4 | | 33  48  13  6 |
| IL6 | rs1800795 | GG  GC  CC  Missing | 52.8 (15.6)  53.1 (15.5)  53.2 (15.0)  54.6 (15.6) | 31  46  16  7 | | 31  47  15  7 | | 33  42  18  7 | | 29  47  17  7 | | 31  43  17  9 | | 30  46  15  8 | | 30  44  16  10 |
| IL8 | rs4073 | TT  TA  AA  Missing | 52.6 (15.6)  53.4 (15.4)  52.9 (15.3)  54.6 (15.4) | 31  45  19  5 | | 29  45  19  6 | | 27  46  21  5 | | 28  46  19  7 | | 27  47  20  6 | | 27  47  18  7 | | 24  48  20  8 |
| IL10 | rs1800871 | CC  CT  TT  Missing | 53.2 (15.3)  53.0 (15.6)  52.9 (15.8)  54.1 (15.7) | 51  37  5  7 | | 54  34  5  7 | | 54  35  5  6 | | 55  34  5  7 | | 54  34  5  7 | | 54  35  4  7 | | 51  35  6  9 |
| IL10 | rs1800872 | CC  CA  AA  Missing | 53.1 (15.4)  52.9 (15.6)  52.8 (15.9)  55.1 (15.0) | 52  38  5  5 | | 53  34  6  6 | | 54  35  5  6 | | 53  34  5  8 | | 54  33  5  7 | | 53  35  4  8 | | 47  35  7  10 |
| IL10^†^ | rs1800890 | AA  AT  TT  Missing | 52.9 (15.5)  52.6 (15.5)  53.8 (15.2)  57.4 (15.6) | 38  47  12  3 | | 38  46  14  2 | | 36  48  14  2 | | 39  44  14  3 | | 36  44  16  4 | | 36  46  14  4 | | 40  36  16  7 |
| IL10^†^ | rs1800896 | AA  AG  GG  Missing | 53.0 (15.5)  52.7 (15.5)  53.7 (15.2)  56.1 (15.8) | 28  51  19  2 | | 27  49  21  2 | | 27  49  22  2 | | 28  47  22  3 | | 27  47  23  4 | | 26  47  23  4 | | 31  43  22  4 |
| IL13 | rs20541 | GG  AG  AA  Missing | 52.8 (15.3)  53.2 (15.6)  52.7 (15.3)  57.4 (16.3) | 64  30  3  3 | | 65  28  4  2 | | 67  28  4  2 | | 63  30  4  3 | | 65  29  3  3 | | 62  29  4  5 | | 55  32  4  8 |
| IL13 | rs1800925 | CC  CT  TT  Missing | 53.0 (15.4)  52.8 (15.6)  52.9 (15.0)  56.5 (15.6) | 62  32  3  3 | | 63  31  4  2 | | 62  32  4  2 | | 62  31  4  3 | | 64  30  3  3 | | 61  31  5  4 | | 60  31  2  7 |
| IL18 | rs187238 | CC  CG  GG  Missing | 53.1 (15.5)  52.6 (15.3)  53.0 (15.9)  57.8 (15.3) | 52  38  7  2 | | 52  40  7  2 | | 52  40  6  2 | | 51  40  6  3 | | 52  38  7  4 | | 53  37  7  4 | | 58  31  5  7 |
| IL18 | rs1946518 | GG  GT  TT  Missing | 53.3 (15.6)  52.6 (15.2)  52.7 (15.7)  58.3 (15.5) | 36  45  16  3 | | 35  48  15  2 | | 34  49  15  2 | | 35  47  15  3 | | 35  46  14  5 | | 37  42  15  6 | | 40  39  12  8 |
| LTA | rs2857713 | TT  CT  CC  Missing | 52.5 (15.4)  52.9 (15.5)  53.2 (15.5)  56.3 (15.2) | 51  36  6  7 | | 51  35  8  7 | | 51  35  7  7 | | 49  35  7  9 | | 47  35  7  11 | | 46  34  8  13 | | 43  36  6  15 |
| LTA | rs3093543 | AA  AC  CC  Missing | 53.0 (15.5)  52.9 (15.1)  0  55.4 (16.1) | 86  12  0  2 | | 83  14  0  2 | | 84  13  0  2 | | 85  13  0  2 | | 83  14  0  3 | | 84  12  0  4 | | 86  10  0  4 |
| LTA | rs1041981 | CC  AC  AA  Missing | 53.0 (15.5)  53.1 (15.4)  52.0 (15.2)  56.3 (15.7) | 41  43  13  3 | | 42  42  13  3 | | 44  42  11  3 | | 43  41  12  4 | | 42  44  11  4 | | 42  42  10  5 | | 44  40  9  8 |
| LTA | rs909253 | TT  TC  CC  Missing | 52.9 (15.5)  53.1 (15.4)  52.1 (15.3)  56.9 (15.5) | 41  43  13  3 | | 42  43  13  3 | | 43  42  12  3 | | 42  42  12  3 | | 41  44  11  4 | | 42  43  10  5 | | 42  40  11  7 |
| MPO | rs2243828 | AA  AG  GG  Missing | 53.0 (15.4)  52.9 (15.5)  52.2 (16.1)  57.0 (15.6) | 59  33  5  3 | | 57  36  5  2 | | 61  33  4  2 | | 60  32  5  3 | | 59  33  4  4 | | 57  34  4  5 | | 57  30  6  7 |
| MPO | rs2333227 | CC  CT  TT  Missing | 52.9 (15.4)  52.6 (15.5)  52.4 (16.0)  56.7 (15.4) | 58  31  4  7 | | 55  35  5  5 | | 59  32  3  5 | | 57  31  5  8 | | 57  30  3  10 | | 53  32  4  11 | | 55  28  5  12 |
| NOS2A^†^ | rs2297518 | GG  AG  AA  Missing | 53.0 (15.4)  52.6 (15.4)  54.7 (15.4)  57.5 (16.0) | 65  29  3  3 | | 63  31  3  2 | | 65  30  4  2 | | 64  29  4  3 | | 64  28  4  4 | | 61  29  5  4 | | 63  23  4  9 |
| NOS3 | rs1799983 | GG  GT  TT  Missing | 52.6 (15.4)  52.9 (15.4)  52.5 (15.5)  57.6 (15.4) | 43  41  11  5 | | 44  40  10  5 | | 43  43  10  4 | | 44  40  10  6 | | 41  40  10  8 | | 40  40  9  11 | | 39  40  9  12 |
| PPARD | rs2016520 | TT  TC  CC  Missing | 53.0 (15.4)  52.8 (15.6)  53.0 (15.4)  56.0 (15.9) | 62  31  3  4 | | 62  31  4  3 | | 63  31  4  3 | | 63  30  3  4 | | 62  30  4  5 | | 63  29  3  5 | | 56  31  5  8 |
| PPARG† | rs709158 | AA  AG  GG  Missing | 52.7 (15.5)  53.1 (15.4)  54.0 (15.5)  55.5 (15.7) | 40  45  11  4 | | 41  43  12  4 | | 41  42  12  5 | | 39  44  12  5 | | 39  44  13  4 | | 38  41  14  7 | | 34  44  15  7 |
| PPARG | rs1175543 | AA  AG  GG  Missing | 52.8 (15.5)  53.1 (15.4)  53.9 (15.4)  54.4 (16.2) | 41  44  10  5 | | 41  42  13  4 | | 41  43  12  5 | | 39  44  12  5 | | 39  44  13  4 | | 39  41  13  7 | | 36  42  15  7 |
| PPARG | rs1801282 | CC  CG  GG  Missing | 52.8 (15.5)  53.1 (15.3)  54.2 (14.7)  57.6 (15.7) | 75  21  1  3 | | 80  16  1  2 | | 77  20  1  2 | | 76  18  2  4 | | 73  20  2  5 | | 75  18  1  6 | | 74  16  1  8 |
| PPARG | rs4684847 | CC  CT  TT  Missing | 53.0 (15.6)  53.3 (15.4)  54.4 (14.4)  54.9 (14.5) | 75  20  1  4 | | 77  17  1  5 | | 76  18  1  5 | | 74  19  2  6 | | 73  20  2  6 | | 75  18  1  6 | | 74  18  1  7 |
| PPARGC1 | rs8192678 | GG  GA  AA  Missing | 52.3 (15.6)  53.1 (15.4)  53.1 (15.0)  57.6 (15.9) | 45  42  10  4 | | 46  42  10  2 | | 44  41  12  3 | | 42  44  11  4 | | 43  42  11  5 | | 41  42  11  6 | | 42  41  8  9 |
| TNF | rs1799724 | CC  CT  TT  Missing | 53.0 (15.4)  52.8 (15.7)  50.1 (14.6)  56.6 (15.8) | 80  16  1  2 | | 82  15  1  2 | | 79  18  1  2 | | 81  16  1  3 | | 82  15  1  3 | | 80  16  1  3 | | 76  18  0  5 |
| TNF | rs1799964 | TT  TC  CC  Missing | 52.9 (15.4)  53.1 (15.6)  53.3 (15.3)  56.8 (15.6) | 62  31  4  3 | | 60  33  5  2 | | 62  32  5  2 | | 59  33  5  3 | | 59  32  5  3 | | 58  33  5  4 | | 58  34  4  5 |
| TNF | rs1800629 | GG  GA  AA  Missing | 53.0 (15.6)  52.9 (15.4)  53.0 (15.6)  55.8 (14.7) | 68  27  2  3 | | 65  28  2  4 | | 67  25  3  5 | | 66  26  3  5 | | 67  25  3  5 | | 65  26  3  6 | | 67  23  2  7 |

^*^ p=0.01 ^†^ p=0.04 for a comparison across groups defined by genotypes using ANOVA (missing category not considered).
