# Supplementary material for: Frequencies of single nucleotide polymorphisms in genes regulating inflammatory responses in a community-based population
Source: BMC Genet. 2007 Mar 14;8:7. doi: 10.1186/1471-2156-8-7 (PMC1838428; doi:10.1186/1471-2156-8-7)
Supplement: Additional File 3 — Table 4. Genotype frequencies by sex in Odyssey and CLUE II subcohort; description: Genotype frequencies in men and women of the combined Odyssey and CLUE II subcohort. [file 1471-2156-8-7-S3.doc]

**Table 4. Genotype frequencies by sex in Odyssey and CLUE II subcohort**

| **Gene** | **SNP** | **Genotype** | **Women (n=6,131)**  **%** | | **Men (n=3,829)**  **%** |
| --- | --- | --- | --- | --- | --- |
| CCR2 | rs1799864 | GG  GA  AA  Missing | 79  14  0.5  6 | 79  14  0.8  6 | |
| CCR5 | rs333 | GG  G-  --  Missing | 75  17  1  7 | 75  17  1  6 | |
| COX1 | rs3842787 | CC  CT  TT  Missing | 82  11  0.5  6 | 83  12  0.6  4 | |
| COX2 | rs5275 | TT  TC  CC  Missing | 41  40  11  7 | 41  42  11  6 | |
| COX2 | rs2143416 | AA  AC  CC  Missing | 66  26  3  5 | 66  26  2  4 | |
| COX2 | rs2206593 | CC  CT  TT  Missing | 84  11  0.4  4 | 85  11  0.3  4 | |
| COX2 | rs2745557 | CC  CT  TT  Missing | 65  27  3  5 | 64  28  3  4 | |
| CRP | rs1205 | CC  CT  TT  Missing | 44  40  10  5 | 43  42  10  4 | |
| CRP ^*^ | rs1800947 | CC  CG  GG  Missing | 76  8  0.4  15 | 78  9  0.7  12 | |
| CRP | rs1130864 | CC  CT  TT  Missing | 45  39  9  6 | 42  40  9  5 | |
| CRP | rs2794521 | TT  TC  CC  Missing | 49  37  8  6 | 50  36  7  5 | |
| CSF1 | rs1058885 | TT  TC  CC  Missing | 37  43  12  7 | 36  44  13  6 | |
| CSF2 | rs1469149 | AA  AC  CC  Missing | 33  47  17  3 | 33  48  16  2 | |
| CSF2 | rs25882 | TT  TC  CC  Missing | 58  30  4  7 | 59  31  4  6 | |
| IFGN | rs2069705 | AA  AG  GG  Missing | 42  41  9  7 | 43  41  10  6 | |
| IL1A | rs1800587 | GG  GA  AA  Missing | 49  38  8  5 | 49  38  8  5 | |
| IL1A | rs17561 | CC  AC  AA  Missing | 49  38  8  5 | 49  38  8  4 | |
| IL1B | rs16944 | CC  CT  TT  Missing | 40  38  11  11 | 40  37  11  12 | |
| IL1B | rs1143634 | CC  CT  TT  Missing | 52  30  5  13 | 52  30  5  13 | |
| IL2 ^†^ | rs2069762 | AA  AC  CC  Missing | 48  37  9  6 | 46  41  8  5 | |
| IL4 | rs2243250 | CC  CT  TT  Missing | 70  21  3  6 | 69  22  2  6 | |
| IL6 | rs1800797 | GG  GA  AA  Missing | 33  47  16  3 | 34  47  17  2 | |
| IL6 | rs1800795 | GG  GC  CC  Missing | 30  45  16  8 | 31  45  17  7 | |
| IL8 | rs4073 | TT  TA  AA  Missing | 27  46  19  6 | 28  46  20  6 | |
| IL10 | rs1800871 | CC  CT  TT  Missing | 53  35  5  7 | 54  34  5  7 | |
| IL10 | rs1800872 | CC  CA  AA  Missing | 53  35  5  7 | 54  34  5  7 | |
| IL10 ^‡^ | rs1800890 | AA  AT  TT  Missing | 35  46  15  4 | 38  45  14  3 | |
| IL10 | rs1800896 | AA  AG  GG  Missing | 28  47  23  3 | 28  48  22  3 | |
| IL13 | rs20541 | GG  GA  AA  Missing | 64  29  4  3 | 64  29  4  3 | |
| IL13 | rs1800925 | CC  CT  TT  Missing | 63  30  4  3 | 62  31  4  3 | |
| IL18 | rs187238 | CC  CG  GG  Missing | 52  39  6  3 | 52  39  6  3 | |
| IL18 | rs1946518 | GG  GT  TT  Missing | 34  46  15  4 | 35  47  14  3 | |
| LTA | rs2857713 | TT  TC  CC  Missing | 48  34  7  10 | 49  35  7  9 | |
| LTA | rs3093543 | AA  AC  CC  Missing | 84  13  0  2 | 84  13  0  3 | |
| LTA | rs1041981 | CC  CA  AA  Missing | 42  43  11  4 | 42  42  12  3 | |
| LTA | rs909253 | TT  TC  CC  Missing | 42  43  11  4 | 42  42  12  3 | |
| MPO | rs2243828 | AA  AG  GG  Missing | 59  33  4  4 | 59  33  4  4 | |
| MPO | rs2333227 | CC  CT  TT  Missing | 57  31  4  9 | 57  32  4  7 | |
| NOS2A | rs2297518 | GG  GA  AA  Missing | 64  28  4  3 | 63  30  4  3 | |
| NOS3 | rs1799983 | GG  GT  TT  Missing | 42  40  10  7 | 42  41  10  6 | |
| PPARD | rs2016520 | TT  TC  CC  Missing | 62  30  3  4 | 63  30  3  4 | |
| PPARG | rs709158 | AA  AG  GG  Missing | 40  43  12  5 | 39  43  13  5 | |
| PPARG | rs1175543 | AA  AG  GG  Missing | 39  43  12  5 | 40  43  13  5 | |
| PPARG ^§^ | rs1801282 | CC  CG  GG  Missing | 75  17  1  4 | 76  18  2  4 | |
| PPARG ^¶^ | rs4684847 | CC  CT  TT  Missing | 73  20  1  6 | 75  18  1  5 | |
| PPARGC1 | rs8192678 | GG  GA  AA  Missing | 43  42  11  4 | 43  42  11  4 | |
| TNF ^†^ | rs1799724 | CC  CT  TT  Missing | 79  17  0.7  3 | 81  15  1  2 | |
| TNF | rs1799964 | TT  TC  CC  Missing | 59  32  5  3 | 60  32  5  3 | |
| TNF | rs1800629 | GG  GA  AA  Missing | 66  26  3  5 | 66  26  2  5 | |

^*^ p=0.05, ^†^ p=0.007, ^‡^ p=0.02, ^§^ p=0.03, ^¶^ p=0.01 from χ^2^ test (missing category not considered)
